# Individual and Population Level Impact of Key HIV Risk Factors on HIV Incidence Rates in Durban, South Africa

**DOI:** 10.1371/journal.pone.0153969

**Published:** 2016-04-22

**Authors:** Gita Ramjee, Suri Moonsamy, Nathlee Samantha Abbai, Handan Wand

**Affiliations:** 1 HIV Prevention Research Unit, Medical Research Council, Durban, South Africa; 2 Department of Epidemiology and Population Health, London School of Hygiene & Tropical Medicine, London, United Kingdom; 3 Department of Global Health, School of Medicine, University of Washington, Washington DC, United States of America; 4 National Center for HIV Epidemiology and Clinical Research, Sydney, Australia; University of Texas Health Science Center San Antonio Texas, UNITED STATES

## Abstract

We aimed to estimate the individual and joint impact of age, marital status and diagnosis with sexually transmitted infections (STIs) on HIV acquisition among young women at a population level in Durban, KwaZulu-Natal, South Africa. A total of 3,978 HIV seronegative women were recruited for four biomedical intervention trials from 2002–2009. Point and interval estimates of partial population attributable risk (PAR) were used to quantify the proportion of HIV seroconversions which can be prevented if a combination of risk factors is eliminated from a target population. More than 70% of the observed HIV acquisitions were collectively attributed to the three risk factors: younger age (<25 years old), unmarried and not cohabiting with a stable/regular partner and diagnosis with STIs. Addressing these risks requires targeted structural, behavioural, biomedical and cultural interventions in order to impact on unacceptably high HIV incidence rates among young women and the population as a whole.

## Introduction

Southern Africa is at the epicenter of the global HIV epidemic, with an estimated 40% of the global HIV burden occurring among individuals in this region [1,2]. One of the key population groups with high reported HIV incidence rates are adolescent girls and young women of between15-24 years of age, who contribute to almost one third of all new reported HIV incident infections [3]. In South Africa (SA) alone, it is reported that greater than 100,000 infections occur in young women each year, which is more than 4 times the number of new reported HIV infections estimated to occur in adolescent and young men [1,2].

The province of KwaZulu-Natal (KZN) in SA, is the region most affected by HIV and AIDS and women account for greater than 60% of infections [4,5]. The most dominant mode of transmission in this hyper-endemic (occurrence of concentrated epidemics in generalized epidemic settings) setting is mainly through unprotected heterosexual contacts.

Combined with behavioral risks such as multiple and concurrent partnerships, lack of condom use and age disparate relationships, independent associations have been reported between HIV and sexually transmitted infections (STIs) including *Chlamydia trachomatis*, *Neisseria gonorrhoeae*, Syphilis and *Trichomonas vaginalis* [6–9]. While biological, behavioral, cultural, socio-economic and structural risk factors have been associated with HIV seroconversion in South African women, the relative contributions of these risk factors vary widely between studies and across different sub-populations [10,11].

In order to inform and establish more effective prevention strategies targeted at young women, there is a need to better understand HIV transmission dynamics and the risks associated with HIV seroconversion.

Estimating an individuals’ risk is the most common way to assess causality, but they do not provide necessary information about their potential impact on HIV incidence rates at a population level. The population attributable risk (PAR) is an epidemiologic measure that serves to provide such information as a quantitative assessment of the potential reduction in disease occurrence if the risk factor were to be entirely removed from the population [12]. PAR provides information about the public health significance of specific and potentially modifiable risk factor(s) on a disease by accounting for both the strength of the association on the outcome of interest, as well as the prevalence of the risk factor in the population. Therefore, identification of measures of attributable risk is imperative not only in guiding policy and prioritizing prevention strategies, but also allows for modifying common risk factors in populations with relative high hazard ratios to assist in minimizing the burden of diseases associated with HIV infection.

In order to advance our understanding of the high incidence rates in young women, we undertook a sub-analysis by combining data from large randomized controlled trials (RCTs) or studies conducted by us to estimate the relative and attributable risks associated with HIV seroconversion. We particularly focused on one unmodifiable risk factor (age) and two modifiable risk factors (not being married and/or cohabiting with a stable/regular partner), and being diagnosed with at least one sexually transmitted disease. Furthermore, this sub-analysis study is one of the first to narrow down individual and combination of multiple risk factors into three specific modifiable factors to address population level impact of such risks on HIV incidence rates among young women.

## Methods

### Study Population

We combined data of 3,978 sexually active women who consented to screening for four studies in the Province of KZN: Methods for Improving Reproductive Health in Africa (MIRA) RCT using the vaginal diaphragm for HIV prevention (September2002-September 2005; undertaken in Umkomaas and Botha’s Hill, South and North East of Durban, respectively) [13]; The Microbicides Development Programme (MDP) Feasibility Study in Preparation for Phase III Microbicide Trials (August 2002-December 2004; undertaken in semi-rural Tongaat and Verulam, northern Durban) [14], the HIV Prevention Trials Network (HPTN) Site Preparedness study (HPTN 055) for Future Implementation of Phase2/IIb/III clinical trials (June 2003—October 2005; undertaken in rural district of Hlabisa, and urban Durban) [6] and the Carraguard® RCT which tested the potential microbicide, Carraguard^®^ [15] at a urban center, Isipingo between (March 2004 –March 2007).

All study populations have previously been described elsewhere [6,13–15]. For the MIRA and HPTN 055 studies, HIV diagnostic testing was determined by using two rapid tests on whole blood sourced from either finger-prick or venipuncture: Determine HIV-1/2 (Abbot Laboratories, Tokyo, Japan) and Oraquick (Orasure Technologies, Bethlehem, PA, USA). The Abbot IMX ELISA test (Abbot Diagnostics, Africa Division) was used for HIV diagnosis during the MDP Feasibility study. For the Carraguard^®^ RCT, HIV serostatus was confirmed with parallel HIV-1 rapid tests and positive/discordant tests were confirmed by third-generation enzyme immunoassay or polymerase chain reaction (PCR) for the detection of HIV-1 ribonucleic acid (RNA).

Briefly, the main eligibility criteria were similar for all studies and included: being sexually active; HIV negative status at screening; willingness to provide written consent and follow study procedure; not pregnant with intention to maintain non-pregnant status; and residence in and around the study area for a minimum of 1 year. At all visits, participants received risk reduction counseling and access to condoms. Counselors emphasized that condoms are the only known method to prevent HIV and STIs, and that condoms should be used for every sex act. Women who were HIV-positive at screening were referred to local health care facilities for care and support. Women who seroconverted during the trials remained in the study and were provided with ongoing counseling and referral to local health care facilities for further care upon completion of the studies. All protocols and informed consent forms were approved by the Biomedical Research Ethics Committee (BREC) at the University of KwaZulu-Natal. All participants provided written informed consent to participate in the studies.

### Statistical Analysis

We conducted prospective observational analysis by combining the data from the four studies as described above. Cox proportional regression models of HIV seroconversion on the discrete time scale of monthly and quarterly visits. With this approach, the behavior variables were assessed at the same time that the blood was drawn for seroconversion testing, and refer to the previous 1-month (for monthly visits) or 3-month time period (for quarterly visits).

Given that none of the study interventions showed efficacy in preventing HIV transmission, we combined the data as one cohort irrespective of treatment arm allocations.

The primary outcome was incident HIV infection as defined time from enrollment to seroconversion, on the basis of a discrete time scale determined by an individual’s monthly /quarterly visit. For women who seroconverted, the time of seroconversion was defined as the time of first positive HIV test result. For cases in which one or more visits were missed in the intervals between the last negative and first positive tests, the time of seroconversion was assumed to be the visit containing the midpoint between these two time points.

### Assessment of modifiable and non-modifiable risk factors

Risk factors were classified as (at least theoretically) modifiable (by public health intervention) and non-modifiable (even theoretically) and/or background risk factors (all persons similarly exposed or potential confounders for HIV transmission). Among the established risk factors, cohabitation status (unmarried and not cohabiting with a stable/regular partner), and incidence of STIs (*Chlamydia trachomatis*, *Neisseria gonorrhoeae*, Syphilis or *Trichomonas vaginalis*) during the follow up were both considered to be potentially modifiable risk factors. Age at infection was considered to be a non-modifiable and/or background risk factor and assumed to remain unchanged.

### Estimation of relative and population attributable risks

Hazard ratios and 95% confidence intervals (95% CIs) for HIV incidence rates were calculated using Cox regression models.

PAR was calculated by combining the hazard ratios and the observed prevalence of the risk factors of interest [16]. Briefly, PAR is used to measure the reduction expected in the number of HIV infected women if all the known risk factors were eliminated from the target population. We refer to this as the *full* PAR (*PAR*_*F*_). In evaluating preventive interventions in a multi-factorial disease settings such as HIV, the primary interest is in the percentage of cases associated with the modifiable risk factors while other non-modifiable risk factors, such as age is kept unchanged. Therefore, we focus here on the *partial* PAR (*PAR*_*p*_) which estimates the percent of cases associated with modifiable risk factors only. Briefly, PAR and their 95% CIs were calculated for individual risk factors as well as their combinations using Cox regression models for the incidence rate of HIV seroconversion and observed prevalence rates of the risk factors of interest.

We also created a “highest risk” category for the women who were “younger than 25 years of age”, “unmarried and not cohabiting with a stable/regular partner” and “diagnosed with other STIs”. Population attributable risks, which were estimates of the proportion of HIV seroconversions during follow-up that would not have occurred if all women had been in “low risk” category, assuming that the observed associations represent causal effects. For simplicity and increased statistical efficiency, we used a single binary categorical variable to calculate the PAR% pertaining to the impact of low-risk factor.

Since age was a strong determinant risk factor of seroconversion, we also stratified our analysis by age groups.

Analyses were performed using SAS statistical software, version 10 (SAS Inc., Cary, NC).

## Results

### Study population

Approximately 41% of women in the study population (n = 3978) were younger than 25 years of age. More than 70% of women were unmarried and not cohabiting with a stable/regular partner and approximately one-quarter of the women (26%) were diagnosed with at least one STI [i.e. (*Chlamydia trachomatis*, *Neisseria gonorrhoeae*, Syphilis or *Trichomonas vaginalis*)] during study follow-up visits. Furthermore, 33% of women reported having lower than secondary education status and 63% reported using at least one type of contraceptive.

Approximately half (50%) of the study population reported no condom use in last sexual acts, whilst slightly just over 50% of them reported more than 3 sexual acts per week. In the overall study population, pregnancy incidence of 18% (data not shown) was observed, whereas a total of 318 women seroconverted for HIV (10% in MIRA trial, 8% in Carraguard trial and 6% in feasibility and HPTN055 studies, respectively).

### Risk factors and their joint impacts of on HIV seroconversion

A total of 318 HIV seroconversions (incidence cases) were observed during study follow-up visits with an overall incidence rate of 6.3 per 100 women-years [95% Confidence interval (CI): 5.6,7.04]. Table 1 provides the frequency distributions and the crude HIV incidence rates for each of the three risk factors in this sub-analysis: younger than 25 years of age, unmarried and not cohabiting with a stable/regular partner and being diagnosed with an STI. HIV seroconversions were significantly higher among younger women as compared to older women (9.63 per 100 person year, 95% CI: 8.35, 11.11 and 4.16 per 100 person year, 95% CI: 3.50, 4.95, p-value<0.001). Women who were unmarried and not cohabiting with a stable/regular partner also had the highest HIV incidence rates with 7.80 per 100 person year (95%CI: 7.00, 8.76) compared to those married or cohabiting with their sexual partners (2.00 per 100 person year, 95% CI: 1.36, 2.94, p-value <0.001). Women who reported being diagnosed with at least one or more STIs (i.e. *Chlamydia trachomatis*, *Neisseria gonorrhoeae*, Syphilis or *Trichomonas vaginalis*) during the study were also significantly associated with HIV seroconversion compared to those diagnosed with no STIs (10.19 per 100 person year, 95% CI: 8.62,12.06 vs. 4.90 per 100 person-year, 95% CI: 4.22,5.66, p-value<0.001).

**Table 1:** Crude incidence rates for HIV seroconversion.

Risk factors	N (%)	Incidence rate (95% CI)	p-value
**Age groups**			<0.001
25+ years	2,337 (57.75)	4.16 (3.50,4.95) per 100 person-year	
<25	1,641 (41.25)	9.63 (8.35,11.11) per 100 person-year	
**Marital Status**			<0.001
Married	947 (23.81)	2.00 (1.36,2.94) per 100 person-year	
Unmarried and not cohabiting with a stable/regular partner	3,031 (76.19)	7.80 (7.00,8.76) per 100 person-year	
**Diagnosis with STI(s)**			<0.001
No	2,925 (73.53)	4.90 (4.22,5.66) per 100 person-year	
Yes	1,053 (26.47)	10.19 (8.62,12.06) per 100 person-year	

Table 2 provides adjusted (for level of education, average number of sexual acts for last 2 weeks, and contraceptive use) hazard ratios (HRs) for these reported risk factors. We presented estimates of the proportion of HIV incidence cases attributable to the three main non-modifiable/modifiable risk factors and possible intervention scenarios for HIV transmission. In the overall study population, three factors, namely, younger age, unmarried and not cohabiting with a stable/regular partner and diagnosis with STIs were associated with 71% of all reported HIV seroconversions (95% CI: 68, 73). Their partial contributions to the HIV seroconversions were 23% (95% CI: 18,27) for those younger than 25 years of age, 59% for those who were unmarried and not cohabiting with a stable/regular partner and 18% for being diagnosed with an STI. In the stratified analysis by age, approximately 50% of young women in the study population (95% CI: 27, 68) and 60% of older women (95% CI: 51, 66) (older) of the reported cases were attributed for those who were unmarried. The high hazard ratios of marital status were responsible for this reported impact among women.

**Table 2:** Individual and population level impacts of risk factors association with HIV seroconversion.

	**Unadjusted Analysis** (individual-level risk)		**Adjusted Analysis†**(individual-level risk)	
**Risk factors**	**HR (95% CI)**	**p-value**	**HR (95% CI)**	**p-value**
**Age groups**				
25+ years	1		1	
<25	2.31(1.84,2.89)	<0.001	1.65 (1.30,210)	<0.001
**Marital Status**				
Married	1		1	
Unmarried and not cohabiting with a stable/regular partner	3.90 (2.60,5.81)	<0.001	2.82 (1.85,4.29)	<0.001
**Diagnosed with/STI**				
No	1		1	
Yes	2.07 (1.66,2.59)	<0.001	1.75 (1.40,2.19)	<0.001
	**Unadjusted Population Attributable Risk** (population-level risk) **OVERALL**		**Adjusted† Population Attributable Risk** (population-level risk) **OVERALL**	
	PAR% (95% CI)		PAR% (95% CI)	
**Unmodifiable risk factor**				
Age <25 years	34 (30,38)		23 (18,27)	
**Modifiable risk factors**				
Unmarried and not cohabiting with a stable/regular partner	68 (63,73)		59 (52,67)	
Diagnoses with STI(s)	22 (19,25)		18 (15,21)	
**FULL PAR†**				
Unmarried and not cohabiting with a stable/regular partner + Diagnosis with STI(s)	73 (68,77)		71 (65,73)	
	**Unadjusted Population Attributable Risk** (population-level risk): **YOUNGER WOMEN (<25 years)**		**Adjusted†Population Attributable Risk** (population-level risk): **YOUNGER WOMEN (<25 years)**	
	PAR% (95% CI)		PAR% (95% CI)	
**Modifiable risk factors**				
Unmarried and not cohabiting with a stable/regular partner	48 (27,69)		42 (21,67)	
Diagnoses with STI(s)	17 (13,21)		16 (13,20)	
**FULL PAR†**				
Unmarried and not cohabiting with a stable/regular partner + Diagnosis with STI(s)	51 (31,71)		48 (27,68)	
	**Unadjusted Population Attributable Risk** (population-level risk) **OLDER WOMEN (25 + years)**		**Adjusted† Population Attributable Risk** (population-level risk) **OLDER WOMEN (25 +years)**	
	PAR% (95% CI)		PAR% (95% CI)	
**Modifiable risk factors**				
Unmarried and not cohabiting with a stable/regular partner	59 (51,66)		57 (49,65)	
Diagnoses with STI(s)	22 (17,26)		20 (16,25)	
**FULL PAR†**				
Unmarried and not cohabiting with a stable/regular partner + Diagnosis with STI(s)	65 (58,72)		61 (53,67)	

**†**level of education, average number of sexual act last 2 weeks, contraceptive use

## Discussion

This is one of the first studies to show that in a combined cohort of women, 71% of observed cases of incident HIV infections among women in Durban, South Africa are associated with one non-modifiable (younger age) and two established modifiable risk factors (unmarried and not cohabiting with a stable/regular partner), and being diagnosed with at least one STI (*Chlamydia trachomatis*, *Neisseria gonorrhoeae*, Syphilis or *Trichomonas vaginalis*) by utilizing PAR as an estimate of the proportion of infections which may have been averted, after taking into account the relationships with other variables. We found that being unmarried and not cohabiting with a stable/regular partner was associated with an almost 3-fold higher risk of HIV seroconversion than being married. As a result, 59% of the population risk was attributable to this behavior. Hence, if this behavior was not present in the population, the number of infections would have been reduced by approximately 60%.

Further, we showed that 26% of women with a diagnosis of an STI was one of the contributing risk factors, the hazard ratio was 1.75 (95% CI: 1.40–2.19) and the PAR was 18%. Although the contribution of an STI is significant, by comparison of the estimated PAR it is clear that being unmarried or not cohabiting with a stable partner, is the stronger driver of population HIV risk than diagnosis with an STI. The situation is particularly grim for women and young girls in sub-Saharan Africa and the burden of disease especially continues to increase among young women. Therefore, an effective intervention is needed, which encourages HIV testing, safe sex counseling, reduction in risky sexual behavior, promoting STI testing and treatment and cohabiting with partners is needed. Should this be achieved, we can expect a reduction in HIV incidence.

In South Africa, significantly high HIV infection rates have been observed among unmarried individuals compared to married individuals [17] and our results are concordant with the reported study. In our setting, women who were unmarried and not cohabiting with a stable/regular partner faced more than a double hazard of HIV acquisition when compared to women that were married or without any sexual partners. The reason why so many women in particular remain unmarried is attributable to cultural practices such as the requirement of high price of “lobola”–where the potential male partner has to provide to the female partner’s family may contribute to lack of formal marriages in this society [18]. In addition, these unmarried women are exposed to structural factors such as migration, transactional sex for economic survival, gender norms and urbanization, which in turn are contributory factors that further augments their vulnerability to HIV [19].

Despite the introduction of HIV prevention and treatment programs, the overall HIV incidence rate of 6.3 per 100 women’s year among women younger than 25 years of age remains unacceptably high as reported in this study. This trend has been observed in the last decade [20–22]. Thus, our findings are consistent with previous reports [20–22]. Our study shows that younger age (<25 years of age) carries a two-fold greater risk of HIV acqusition.

The importance of measuring HIV incidence is key to understanding the dynamics of HIV transmission and acquisition to shape and modify effective responses. The persistence of high HIV incidence rates and the vulnerability of young women in our setting remains a major health concern. It has been suggested that the risk for HIV increases as soon as young women initiates sexual intercourse–biological vulnerability [23]. For instance, in older women, the endocervix is made up of simple columnar epithelium, while the ectocervix and vagina are lined with a stratified squamous epithelium. As women age, the columnar epithelium of the ectocervix transforms into squamous epithelium. However, in young women, cervical ectopy and the presence of a simple columnar epithelium is part of the normal physiology, and this leads to increased susceptibility to HIV and other STIs [23]. In addition, young women have relatively higher levels of genital inflammation, which further increases the risk of acquiring HIV [23].

There are various other socio-behavioural factors that increases a young women’s risk for acquiring HIV such as early age of sexual debut, age-disparate relationships, inconsistent condom use, transactional sex and concurrent sexual partners [24]. Studies have reported that residing in urban informal settlements, being unmarried and/or unemployed was associated with higher HIV incidence underscoring some of the underlying structural and social factors that drive the epidemic [24].

Age disparate relationships were reported to be one of the primary reasons for high HIV incidence rates among young women. The aggregating incidence rates of HIV infections with increasing age implies that a young woman engaging in a sexual relationship with an older man is at a higher risk of acquiring HIV compared to a young woman engaging with a male peer. Dellar and her colleagues (2015) reported that young women less than 25 years of age constitute nearly 30% of all new infections and are more likely to seroconvert 5–7 years earlier compared to their male counterparts [25]. In addition, these very young women are eight times more likely to acquire HIV infections compared to their male counterparts [25]. Furthermore, a young woman engaging in a relationship with an older man may be less likely to negotiate condom use given the gender-power dynamics, which further increases her risk of HIV acquisition.

Conversely, recent data suggests that the epidemic in South Africa may be shifting [26,27]. A more recent study conducted by Street and her colleagues among 1355 women aged 16 years and older in the South African setting reported that no significant correlation exists between sexual partner age disparity and HIV acquisition [26]. Women in this study were divided into three distinct categories: non-age disparate, intra-generational age disparate relationships and inter-generational age disparate relationships. Findings suggest that age, marital status and concurrency of sexual partners were the main contributing factors for inter-generational age disparate relationships between younger women and older men [26]. Similarly, Balkus and her colleagues analyzed data from women that participated in the Vaginal and Oral Interventions to Control the Epidemic (VOICE) clinical trial and reported that no association exists between male partner age and risk of HIV acquisition of women who were involved in age-disparate relationships [27]. Women who reported to be in an age disparate relationship with a male partner older than five or ten years did not appear to be at a high risk of HIV-1 acquisition.

When considering the apparently uniquely high per-act HIV acquisition risk in young women, it is also necessary to consider other relevant contextual factors that may mediate the infection environment such as other STIs and contraceptive use. Many bacterial and viral STIs are associated with increased risk of HIV infection. We show that women who were diagnosed with STIs were at increased risk for HIV infections. Our findings are supported by previous studies in South Africa [28,29][30]. The mechanism by which STIs increase susceptibility to HIV has been described by Kleppa et al [31,32], Reddy et al [33] and Sperling et al [34]. Sexually transmitted infections have been shown to increase susceptibility to HIV, by causing disruption of the genital epithelium, by increasing the number of HIV target cells and by immune activation in the genital mucosa [31]. Furthermore, STIs are the major causes of inflammatory cytokine up-regulation and immune cell recruitment to the genital mucosa [33,34].

Beyond STIs, other biological risk factors may also be amplified in young women. A recent study by Finchorova et al suggests that young women of reproductive age, who use hormonal contraceptives have altered cervical immunity in presence of genital tract infections such as STIs, which contributes to the increased risk to HIV acquisition [35]. Similarly, in one of our earlier reports, we assessed the population-level impact of hormonal contraceptive use (i.e. injectables and pills) in relation to HIV-1 seroconversion and the incidence of pregnancy during follow-up from two combined cohorts of HIV-1 negative, non-pregnant women who participated in two biomedical trials (MDP 301 and Carraguard) conducted in Durban, south Africa [36]. In this prospective cohort study, hormonal contraceptive use was considered a modifiable risk factor of interest, whilst adjusting for all other confounding factors. Approximately 78% of women reported hormonal contraceptive use in the study [36]. A higher proportion of hormonal contraception was reported among young and unmarried women [36]. Women who reported using hormonal contraceptives at enrolment in the trial had a higher risk of HIV-1 seroconversion (adjusted hazards ratio: 1.24; 95% CI: 0.97–1.58) than women who reported using other types of contraceptives at enrolment [36]. At the population level, the use of hormonal contraceptives (pills or injectables) at baseline and during study follow-up accounted for approximately 20% (95% CI: 16–22) of HIV-1 seroconversions. However, the partial PAR indicated a relative impact of 12% (95% CI: 9.0–15.7). On the other hand, 72% (95% CI: 66–77) of the pregnancies could have been avoided if all women had used hormonal contraceptives [36].

The question remains as to how we address the multiple risks that women face in acquiring HIV in KwaZulu-Natal and their contribution to the overall population risk? Biomedical interventions for HIV prevention such as vaginal microbicides did not yield a positive outcome in all but one trial [37,38] and PreExposure prophylaxis (PrEP) for HIV prevention showed conflicting outcomes among women in South Africa [39]. The poor outcomes were associated mainly with low adherence to product use. This may be in part due to women in SA who do not perceive themselves to be at risk of HIV acquisition [40,41]. For example, the FEM-PrEP HIV Prevention trial was unable to demonstrate the effectiveness antiretroviral (ARV) agent Truvada, among women at high risk for HIV exposure [41]. A sub-analysis of the FEM-PrEP trial data was conducted to assess whether low perceived HIV risk could be a factor for poor adherence. Women were assessed for perceived HIV risk at enrolment and follow-up visits for a period of four weeks. Women who perceived themselves to be at HIV risk were better adherers to the study product compared to those women who did not perceive themselves at risk [41]. An array of factors such as concurrency of partners, sexual contacts without condom use and knowledge of partner HIV status were associated with higher risk perception [41]. These results indicated a significant correlation between the degree of risk perception and level of adherence. Another study (unpublished Ramjee et al) suggests that women, who had perceived themselves at risk of pregnancy and were using condoms for pregnancy prevention, were more likely to adhere to additional prevention options such as a vaginal microbicide. These findings suggest that risk perceptions may be a key element in addressing adherence to study products for not only PreExposure prophylaxis (PrEP), but other HIV prevention options. Given the high incidence rates, it is counter intuitive that women in our setting do not perceive themselves at risk of HIV.

Structural interventions such as cash transfer initiatives that targeted young women in South Africa did not have an impact on HIV incidence [42,43]. Health systems at primary health care centres are able to treat STIs. However, we need to understand if women do seek these treatments and whether if these healthcare systems are integrated in the primary holistic care to women [43].

It is evident from our study that a woman’s perceived risk and her actual risk may differ. Hence, we show that there are other risk factors to HIV acquisition and not just sexual behaviour. Assessment of her social status, sexual behaviour patterns, role of the male partner and his HIV and migratory status, type of relationship between sexual partners (to rule out gender based violence) and her health seeking behaviour regarding regular HIV/STI testing and reproductive care is critical. Current data shows that we are unlikely to have a “one-size fits all” prevention package, but based from collective insights through numerous research studies in our setting; we know that addressing the epidemic among young women in KwaZulu-Natal will require an integrated effort at all levels.

## Conclusion

To our knowledge, this is the first study to investigate the PAR of HIV risk factors in Durban, South Africa. Our results imply that over 70% of the observed HIV seroconversions which were collectively attributed to three risk factors: younger age (<25 years old), unmarried and diagnosis with STIs could be avoided by implementing targeted combinations of behavioural, structural, biomedical and cultural interventions. The most efficient use of scarce resources in reducing HIV infections will require complex balancing between the PAR for a given risk factor(s), the efficacy of interventions to modify the risk factor, and the cost of these interventions. In KZN, we need to address a combination of factors including biological, behavioral, structural and cultural issues in order to reduce HIV infection rates among women and the South African population as a whole. It requires a collective effort from all levels of society including policy makers, traditional and community leaders, community organizations, health systems, researchers, and organizations specifically targeting women’s health in the country.

## References

[1] UNAIDS (2013) Global Report on the Global AIDS epidemic Geneva, Switzerland: Joint United Nations Program on HIV/AIDS (UNAIDS).

[2] Plan. NS (2011) National Strategic Plan on HIV, STIs and TB:2012–2016, Department of Health, South Africa

[3] UNAIDS (2012) Every minute, a young woman is infected with HIV Geneva, Switzerland: Joint United Nations Program on HIV/AIDS (UNAIDS).

[4] Shisana O, Rehle T, Simbayi LCea (2009) South African National HIV Prevalence, Incidence, Behaviour and Communication Survey 2008: A Turning tide among teenagers? Cape Town: HSRC Press.

[5] Abdool Karim SS, Churchyard GJ, Karim QA, Lawn SD (2009) HIV infection and tuberculosis in South Africa: an urgent need to escalate the public health response. Lancet 375: 921–933. 10.1016/S0140-6736(09)60916-8 PMC2803032 19709731

[6] Ramjee G, Williams B, Gouws E, Van Dyck E, De Deken B, Karim SA (2005) The impact of incident and prevalent herpes simplex virus-2 infection on the incidence of HIV-1 infection among commercial sex workers in South Africa. J Acquir Immune Defic Syndr 39: 333–339. 15980695 10.1097/01.qai.0000144445.44518.ea

[7] Losina E, Bassett IV, Giddy J, Chetty S, Regan S, Walensky RP, et al (2010) The “ART” of linkage: pre-treatment loss to care after HIV diagnosis at two PEPFAR sites in Durban, South Africa. PloS one 5: 1–8. 10.1371/journal.pone.0009538 PMC2832018 20209059

[8] Wilkinson D, Connolly AM, Harrison A, Lurie M, Karim SS (1998) Sexually transmitted disease syndromes in rural South Africa. Results from health facility surveillance. J Sex Transm Dis 25: 20–23. 10.1097/00007435-199801000-00005 9437780

[9] Naidoo S, Wand H, Abbai NS, Ramjee G (2014) High prevalence and incidence of sexually transmitted infections among women. AIDS Res Ther 11: 1–7. 25243015 10.1186/1742-6405-11-31 PMC4168991

[10] Mabala R (2006) From HIV prevention to HIV protection: addressing the vulnerability of girls and young women in urban areas. Environ Urban 18: 407–432.

[11] Ramjee G, Daniels B (2013) Women and HIV sub-Saharan Africa. AIDS Res Ther 10: 1–9. 24330537 10.1186/1742-6405-10-30 PMC3874682

[12] Spiegelman D, Hertzmark E, Wand HC (2007) Point and interval estimates of partial population attributable risks in cohort: Examples and software. Cancer Causes Control 18: 571–579. 17387622 10.1007/s10552-006-0090-y

[13] Bassett IV, Regan S, Chetty S, Giddy J, Uhler LM, Holst H, et al (2010) Who starts antiretroviral therapy in Durban, South Africa?… not everyone who should. AIDS (London, England) 24: 37–44. 10.1097/01.aids.0000366081.91192.1c PMC3521614 20023438

[14] Nunn A, McCormack S, Crook AM, Pool R, Rutterford C, Hayes R (2009) Microbicides Development Programme: design of a phase III trial to measure the efficacy of the vaginal microbicide PRO 2000/5 for HIV prevention. Trials 10: 1–12. 19860888 10.1186/1745-6215-10-99 PMC2774685

[15] Skoler-Karpoff S, R G., Ahmed K, Altin iL, Plagianos MG, Friedland B, et al (2008) Efficacy of Carraguard for prevention of HIV infection in women in South Africa: A randomised, double-blind, placebo-controlled trial. Lancet 372: 1977–1987. 10.1016/S0140-6736(08)61842-5 19059048

[16] Wand H, Ramjee G (2011) Combined impact of sexual risk behaviors for HIV seroconversion among women in Durban, South Africa: implications for prevention policy and planning. AIDS Behav 15: 479–486. 10.1007/s10461-010-9845-2 20981479

[17] Shisana O, Rehle T, Simbayi LC, Zuma K, Jooste S, Zungu N, et al (2014) South African National HIV Prevalence, Incidence and Behaviour Survey, 2012 Cape Town, HSRC Press. 10.2989/16085906.2016.1153491 27002359

[18] Ramjee G, Daniels B (2013) Women and HIV in sub-Saharan Africa. AIDS Res Ther 10: 1–9. 24330537 10.1186/1742-6405-10-30 PMC3874682

[19] Jewkes R, Flood M, Lang J (2014) From work with men and boys to changes of social norms and reduction of inequities in gender relations: a conceptual shift in prevention of violence against women and girls. The Lancet 385: 1580–1589. 10.1016/S0140-6736(14)61683-4 25467578

[20] Abdool Karim QA, Kharsany A, Frohlich JA, Werner L, Mashego M, Mlotshwa M, et al (2010) Stabilizing HIV prevalence masks high HIV incidence rates amongst rural and urban women in KwaZulu-Natal, South Africa. Int J Epidemiol 40: 922–930. 10.1093/ije/dyq176 21047913 PMC3156366

[21] Ramjee G, Wand H, Whitaker C, McCormack S, Padian N, Kelly C, et al (2012) HIV incidence among non-pregnant women living in selected rural, semi-rural and urban areas in Kwazulu-Natal, South Africa. AIDS Behav 16: 2062–2071. 21947836 10.1007/s10461-011-0043-7 PMC3458192

[22] Naicker N, Kharsany AM, Werner L, van Loggerenberg F, Mlisana K, Garrett N, et al (2015) Risk Factors for HIV Acquisition in High Risk Women in a Generalised Epidemic Setting. AIDS Behav: 1–12. 25662962 10.1007/s10461-015-1002-5 PMC4506252

[23] Santelli JS, Edelstein ZR, Mathur S, Wei Y, Zhang W, Orr MG, et al (2013) Behavioral, Biological, and Demographic Risk and Protective Factors for New HIV Infections among Youth, Rakai, Uganda. J Acquir Immune Defic Syndr 63: 393 = 400. 10.1097/QAI.0b013e3182926795 23535293 PMC4131841

[24] Wand H, Ramjee G (2012) The relationship between age of coital debut and HIV seroprevalence among women in Durban, South Africa: a cohort study. BMJ open 2: 1–8. 10.1136/bmjopen-2011-000285 PMC3253418 22223838

[25] Dellar R, Dlamini S, Karim Q (2015) Adolescent girls and young women: Key populations for HIV epidemic control. J Int AIDS Soc 18: 64–70. 10.7448/IAS.18.2.19408 PMC4344544 25724504

[26] Street R, Reddy T, Ramjee G (2015) The generational effect on age disparate partnerships and the risk for human immunodeficiency virus and sexually transmitted infections acquisition. Int J STD AIDS: 1–7. 26138899 10.1177/0956462415592325

[27] Balkus J, Nair G, Montgomery E, Mishra A, Palanee-Phillips T, Ramjee G, et al (2015) Age-Disparate Partnerships and Risk of HIV-1 Acquisition among South African Women Participating in the VOICE Trial. J Acquir Immune Defic Syndr. 10.1097/QAI.0000000000000715 PMC4573287 26049280

[28] Naidoo S, Wand H, Abbai NS, Ramjee G (2014) High prevalence and incidence of sexually transmitted infections among women living in Kwazulu-Natal, South Africa. AIDS Res Ther 11: 31 10.1186/1742-6405-11-31 25243015 PMC4168991

[29] Mlisana K, Naicker N, Werner L, Roberts L, van Loggerenberg F, Baxter C, et al (2012) Symptomatic vaginal discharge is a poor predictor of sexually transmitted infections and genital tract inflammation in high-risk women in South Africa. ‎ J Infect Dis 206: 6–14. 10.1093/infdis/jis298 22517910 PMC3490689

[30] Wand H, Ramjee G (2015) Biological impact of recurrent sexually transmitted infections on HIV seroconversion among women in South Africa: results from frailty models. J Int AIDS Soc 18: 1–6. 10.7448/IAS.18.1.19866 PMC4410128 25912181

[31] Kleppa E, Ramsuran V, Zulu S, Karlsen GH, Bere A, Passmore J-AS, et al (2014) Effect of female genital schistosomiasis and anti-schistosomal treatment on monocytes, CD4+ T-cells and CCR5 expression in the female genital tract. PloS one 9. 10.1371/journal.pone.0098593 PMC4045760 24896815

[32] Kleppa E, Ramsuran V, Zulu S, Karlsen GH, Bere A, Passmore J-AS, et al (2014) Effect of female genital schistosomiasis and anti-schistosomal treatment on monocytes, CD4+ T-cells and CCR5 expression in the female genital tract. PloS one 9: 1–9. 10.1371/journal.pone.0098593 PMC4045760 24896815

[33] Reddy B, Rastogi S, Das B, Salhan S, Verma S, Mittal A (2004) Cytokine expression pattern in the genital tract of Chlamydia trachomatis positive infertile women–implication for T‐cell responses. Clin Exp Immunol 137: 552–558. 15320905 10.1111/j.1365-2249.2004.02564.x PMC1809142

[34] Sperling R, Kraus TA, Ding J, Veretennikova A, Lorde-Rollins E, Singh T, et al (2013) Differential profiles of immune mediators and in vitro HIV infectivity between endocervical and vaginal secretions from women with Chlamydia trachomatis infection: A pilot study. J Reprod Immunol 99: 80–87. 10.1016/j.jri.2013.07.003 23993451 PMC3874462

[35] Fichorova R, Chen P, Morrison C, Doncel G, Mendonca K, Kwok C, et al (2015) The Contribution of Cervicovaginal Infections to the Immunomodulatory Effects of Hormonal Contraception. mBio 6: 1–10. 10.1128/mBio.00221-15 PMC4556810 26330510

[36] Ramjee G, Wand H (2012) Population-level impact of hormonal contraception on incidence of HIV infection and pregnancy in women in Durban, South Africa. Bull World Health Organ 90: 785–755. 10.2471/BLT.12.105700 PMC3471058 23109742

[37] Marrazzo JM, Ramjee G, Richardson BA, Gomez K, Mgodi N, Nair G, et al (2015) Tenofovir-Based Preexposure Prophylaxis for HIV Infection among African Women. N Engl J Med 372: 509–518. 10.1056/NEJMoa1402269 25651245 PMC4341965

[38] Abdool Karim Q, Abdool Karim S, Frohlich J, Groble rA, Baxter C, Mansoor L, et al (2010) Effectiveness and safety of tenofovir gel, an antiretroviral microbicide, for the prevention of HIV infection in women. Science 3329: 1168–1174. 10.1126/science.1193748 PMC3001187 20643915

[39] Rees H, Sinead A, Moretlwe D, Lombard C, Baron D, Panchia R, et al. FACTS 001 Phase III Trial of Pericoital Tenofovir 1% Gel for HIV Prevention in Women 2015 February 23–26, 2015; Seattle, Washington.

[40] Van Damme L, Corneli A, Ahmed K, Agot K, Lombaard J, Kapiga S, et al (2012) Preexposure Prophylaxis for HIV Infection among African Women. N Engl J Med 367: 411–422. 10.1056/NEJMoa1202614 22784040 PMC3687217

[41] Corneli A, Wang M, Agot K, Ahmed K, Lombaard J, Van Damme L (2014) Perception of HIV risk and adherence to a daily, investigational pill for HIV prevention in FEM-PrEP. J Acquir Immune Defic Syndr 67: 555–563. 10.1097/QAI.0000000000000362 25393942

[42] Pettifor A, MacPhail C, Selin Aea. HPTN 068 conditional cash transfer to prevent HIV infection among young women in South Africa: results of a randomized controlled trial; 2015 July, 19–22; Vancouver, Canada.

[43] Karim Q, Leask K, Kharsany Aea. Impact of conditional cash incentives on HSV-2 and HIV prevention in rural South African high school students: results of the CAPRISA 007 cluster randomized controlled trial; 2015 July 19–22; Vancouver, Canada.

